# Supplementary material for: Autoamplification and Competition Drive Symmetry Breaking: Initiation of Centriole Duplication by the PLK4-STIL Network
Source: iScience. 2018 Oct 11;8:222–35. doi: 10.1016/j.isci.2018.10.003 (PMC6197440; doi:10.1016/j.isci.2018.10.003)
Supplement: Document S1. Transparent Methods, Figures S1 and S2, and Tables S1 and S2 [file mmc1.pdf]

ISCI, Volume *8*

Supplemental Information

Autoamplification and Competition Drive Symmetry Breaking: Initiation of Centriole Duplication by the PLK4-STIL Network

Marcin Leda, Andrew J. Holland, and Andrew B. Goryachev

Transparent methods

Mathematical formulation of the model

We estimated that the proximal end of a centriole is a cylinder with radius *R = 250nm* and height *h = 400nm* (Lawo et al., 2012; Sonnen et al., 2012) that is immersed into the spatially homogeneous cytoplasm. Rather than assuming that PLK4 and STIL bind to and react on a 2D surface, we postulated that these reactions happen within a volume determined by a cylindrical shell with finite thickness. This choice is motivated by the fact that binding sites for PLK4 are molecularly determined by the N-termini of CEP152 and CEP192, which are thought to be positioned 200-220*nm* and ~300*nm* away from the center of the centriole, respectively (Park et al., 2014; Sonnen et al., 2013; Sonnen et al., 2012). For simplicity, we assumed that these fixed binding sites are approximately uniformly distributed within the reaction volume. We then subdivided it into *N* = 2 or 9 equal compartments (Figure S1B). Therefore, within each compartment, numbered by index *i = 1,2…N,* the concentrations of free PLK4 ($[P_i]$) and PLK4-STIL complexes ($[PS_i]$, $[PS^*_i]$, $[P^*S_i]$, and $[P^*S^*_i]$) were assumed spatially homogeneous. Compartments are connected by the common cytoplasm where the concentrations of free PLK4 ($[P_c]$), free STIL ($[S_c]$) and their complexes ($[PS_c]$, $[PS^*_c]$, $[P^*S_c]$ and $[P^*S^*_c]$) are also spatially homogeneous.

The following six types of reactions take place in every centriole compartment and/or in the cytoplasm:

1) Phosphorylation of PLK4 and STIL within the same PLK4-STIL complex. The corresponding reaction rates in the cytoplasm ($R_{1,c}$, $R_{2,c}$, $R_{3,c}$ and $R_{4,c}$) and on the centriole ($R_{1,i}$, $R_{2,i}$, $R_{3,i}$ and $R_{4,i}$) are given as follows:
$$R_{1,c} = k_1[PS_c], R_{2,c} = k_2[PS_c], R_{3,c} = k_3[PS^*{}_c], R_{4,c} = k_4[P^*S_c]$$
$$R_{1,i} = k_1[PS_i], R_{2,i} = k_2[PS_i], R_{3,i} = k_3[PS^*{}_i], R_{4,i} = k_4[P^*S_i]$$

2) Dephosphorylation of PLK4 and STIL with the reaction rates in the cytoplasm ($R_{-1,c}$, $R_{-2,c}$, $R_{-3,c}$ and $R_{-4,c}$) and on the centriole ($R_{-1,i}$, $R_{-2,i}$, $R_{-3,i}$ and $R_{-4,i}$):
$$R_{-1,c} = k_{-1}[PS^*{}_c],\ R_{-2,c} = k_{-2}[P^*S_c],\ R_{-3,c} = k_{-3}[P^*S^*{}_c],\ R_{-4,c} = k_{-4}[P^*S^*{}_c]$$
$$R_{-1,i} = k_{-1}[PS^*{}_i],\ R_{-2,i} = k_{-2}[P^*S_i],\ R_{-3,i} = k_{-3}[P^*S^*{}_i],\ R_{-4,i} = k_{-4}[P^*S^*{}_i]$$

3) Crossphosphorylation of PLK4 and STIL on the centriole ($R_{5,i}$, $R_{6,i}$, $R_{7,i}$ and $R_{8,i}$).
$$R_{5,i} = k_5[PS^*{}_i][P^*S^*{}_i], R_{6,i} = k_6[P^*S_i][P^*S^*{}_i], R_{7,i} = k_7[PS_i][P^*S^*{}_i],$$
$$R_{8,i} = k_8[PS_i][P^*S^*{}_i]$$

4) Binding/unbinding of free PLK4 and PLK4-STIL complexes from the cytoplasm to the centriole ($R_{10,i}$, $R_{11,i}$, $R_{12,i}$, $R_{14,i}$, $R_{15,i}$ and $R_{16,i}$). Here and in 5) parameters *a* and *b* represent concentration conversion factors arising because molecules move between centriolar compartments and the cytoplasm. Precise values of these parameters depend on the specific reaction and are given below in the formulation of the model equations.
$$R_{10,i}(a,b) = k_{10}[PS_c]a - k_{-10}[PS_i]b,$$
$$R_{11,i}(a,b) = k_{11}[PS^*{}_c]a - k_{-11}[PS^*{}_i]b,$$
$$R_{12,i}(a,b) = k_{12}[P^*S_c]a - k_{-12}[P^*S_i]b,$$
$$R_{14,i}(a,b) = k_{14}[P^*S^*{}_c]a - k_{-14}[P^*S^*{}_i]b,$$

$R_{16,i}(a,b) = k_{16}[P_c]a - k_{-16}[P_i]b$

5) Association/dissociation of free PLK4 and STIL in the cytoplasm ($R_{15,c}$) or on the centriole ($R_{15,i}$).

   $R_{15,c} = k_{15}[P_c][S_c] - k_{-15}[PS_c]$,

   $R_{15,i}(a,b) = k_{15}[P_i][S_c]a * N_i - k_{-15}[PS_i]b$,

   here $N_i = 1 + am * (U(0,1) - 0.5)$ is the molecular noise term, U(0,1) is the uniform distribution between 0 and 1 produced by a standard random number generator, *am < 1* is the noise amplitude.

6) Production of the free PLK4 (P_c) and STIL (S_c) in the cytoplasm (R_{24} and R_{25}, respectively).

   $R_{24} = k_{24}, R_{25} = 0\ if(t < t_{STIL}),\ otherwise\ R_{25} = k_{25}$

7) Protein degradation. Free unphosphorylated PLK4 (P_c) and STIL (S_c) are assumed to slowly degrade in the cytoplasm with reaction rates R_{-24} and R_{-25}, respectively. PLK4-STIL complexes in which PLK4 is phosphorylated (P*S and P*S*) are degraded in the cytoplasm ($R_{26,c}$ and $R_{27,c}$) and *in situ* on the centriole ($R_{26,i}$ and $R_{27,i}$):

   $R_{-24} = k_{-24}[P_c], R_{-25} = k_{-25}[S_c]$

   $R_{26,c} = k_{26,c}[P^*S_c], R_{27,c} = k_{27,c}[P^*S^*{}_c]$

   $R_{26,i} = k_{26}[P^*S_i], R_{27,i} = k_{27}[P^*S^*{}_i]$

7a) In the model with nonlinear degradation, degradation rates $R_{26,i}$ and $R_{27,i}$ are replaced by $R_{26,i} = a_1[P^*S_i] + \frac{a_2[P^*S_i]}{[P^*S_i]^2+a_3^2}, R_{27,i} = a_1[P^*S^*{}_i] + \frac{a_2[P^*S^*{}_i]}{[P^*S^*{}_i]^2+a_3^2}$

The empirical nonlinear second term is formulated in such a way that it approaches 0 at high concentrations of P*S and P*S*. This has an overall effect that the degradation of P*S and P*S* is faster at their lower concentrations to model the protective effect of STIL at high protein densities.

Values of all reaction rate constants k_i are given in the Table S1. The system of ordinary differential equations that describes temporary dynamics of all model species in accordance with the reaction network shown in Figure 1 is as follows:

$$\frac{d[PS_i]}{dt} = -R_{1,i} + R_{-1,i} - R_{2,i} + R_{-2,i} - R_{7,i} - R_{8,i} + R_{10,i}\left(\frac{V_{cyt}}{V}, 1\right) + R_{15,i}\left(\frac{V_{cyt}}{V}, 1\right),$$

$$\frac{d[PS^*{}_i]}{dt} = R_{1,i} - R_{-1,i} - R_{3,i} + R_{-3,i} - R_{5,i} + R_{7,i} + R_{11,i}\left(\frac{V_{cyt}}{V}, 1\right),$$

$$\frac{d[P^*S_i]}{dt} = R_{2,i} - R_{-2,i} - R_{4,i} + R_{-4,i} - R_{6,i} + R_{8,i} - R_{26,i} + R_{12,i}\left(\frac{V_{cyt}}{V}, 1\right),$$

$$\frac{d[P^*S^*{}_i]}{dt} = R_{3,i} - R_{-3,i} + R_{4,i} - R_{-4,i} + R_{5,i} + R_{6,i} - R_{27,i} + R_{14,i}\left(\frac{V_{cyt}}{V}, 1\right),$$

$$\frac{d[P_i]}{dt} = -R_{15,i}\left(\frac{V_{cyt}}{V}, 1\right) + R_{16,i}\left(\frac{V_{cyt}}{V}, 1\right),$$

$$\frac{d[PS_c]}{dt} = -R_{1,c} + R_{-1,c} - R_{2,c} + R_{-2,c} - \sum_{i=1}^{N} R_{10,i}\left(1, \frac{V}{V_{cyt}}\right) + R_{15,c},$$

$$\frac{d[PS^*{}_c]}{dt} = R_{1,c} - R_{-1,c} - R_{3,c} + R_{-3,c} - \sum_{i=1}^{N} R_{11,i}\left(1, \frac{V}{V_{cyt}}\right),$$

$$\frac{d[P^*S_c]}{dt} = R_{2,c} - R_{-2,c} - R_{4,c} + R_{-4,c} - R_{26,c} - \sum_{i=1}^{N} R_{12,i}\left(1, \frac{V}{V_{cyt}}\right),$$

$$\frac{d[P^*S^*{}_c]}{dt} = R_{3,c} - R_{-3,c} + R_{4,c} - R_{-4,c} - R_{27,c} - \sum_{i=1}^{N} R_{14,i}\left(1, \frac{V}{V_{cyt}}\right),$$
$$\frac{d[P_c]}{dt} = R_{24} - R_{15,c} - \sum_{i=1}^{N} R_{16,i}\left(1, \frac{V}{V_{cyt}}\right),$$
$$\frac{d[S_c]}{dt} = R_{25} - R_{15,c} - \sum_{i=1}^{N} R_{15,i}\left(1, \frac{V}{V_{cyt}}\right),$$

here $V_{cyt}/V = 10^{-4}$ is the conversion factor (Milo and Phillips, 2016) due to the transition of molecules between the model compartments and should be substituted into the reaction rates determined in 4) and 5) instead of *a* and *b* in accordance with the above equations. To reduce the model complexity, we assume that only species P*S* can catalyse crossphosphorylation of PLK4 and STIL. However, no qualitative changes in the model behaviour were observed if other PLK4-STIL complexes were also permitted to catalyse the crossphosphorylation reactions. The molecular noise in our model is treated in the approach of chemical Langevin equation (Gillespie, 2000). Unless stated otherwise, noise amplitude in reaction $R_{15,i}$ was set to $am = 10^{-1}$. Application of noise to reactions other than reactions describing binding of proteins from the cytoplasm produces no qualitative differences in the model behavior. Initial concentrations of all species were equal to 0. PLK4 production was initiated at $t = 0$, while STIL production was initiated with the delay $t_{STIL} = 2h$. The system was solved numerically with the explicit super-time-stepping method (Alexiades et al., 1996) using a custom C code. Results of simulations were analyzed and plotted using Matlab (MathWorks, Natick, MA).

Stability analysis of stationary states

Stability analysis of the model symmetric stationary state (such as shown in Figure 3) was performed by numerically solving an eigenvalue problem of the order $5N + 6$. Due to this high order of the characteristic polynomial, the analysis was performed for the system with two components $N = 2$. To compute stability of the steady state on a 2D plane of parameters, as presented in Figure 3, the stationary state for the given values of chosen parameters was first found by the standard Newton method, using the previously computed values as the initial guess. Then, eigenvalues λ_i were computed using the Arnoldi iteration method (Arnoldi, 1951). The region where max(Re(λ_i)) is positive is shown in Figure 3 by color.

Loss of symmetry breaking in the model without competition

The system without communication between the centriolar compartments and, thus, competition, is defined by the following equation

$$k_{-10} = k_{-11} = k_{-12} = k_{-14} = k_{-15} = k_{-16} = 0, \quad [1]$$

i.e. reaction rate constants of dissociation of all model species from the centriole into the cytoplasm are set to zero as described in the main text. Substituting [1] into the model equations at the steady state one can readily obtain that the stationary concentration of the free PLK4 in all centriolar compartments are equal to

$$[P_i]_{SS} = \frac{k_{16}[P_c]_{SS}}{k_{15}[S_c]_{SS}}, \quad [2]$$

which implies that, regardless of the number of compartments, they all have equal concentration of free PLK4 that is determined only by the cytoplasmic concentrations of PLK4 and STIL. This is in contrast with the full model in which recycling of molecules back to the cytoplasm is enabled:

$$[P_i]_{SS} = \frac{k_{16}[P_c]_{SS} + k_{-15}V/V_{cyt}[PS_i]_{SS}}{k_{15}[S_c]_{SS} + k_{-16}V/V_{cyt}}. \qquad [3]$$

As seen from equation [3], in this case, the concentration of free PLK4 is dependent on the concentration of PLK4-STIL in the same compartment and, thus, can be distinct for each model compartment. Furthermore, at the steady state the total degradation flux of STIL in each compartment,

$$k_{26}[P^*S_i]_{SS} + k_{27}[P^*S^*{}_i]_{SS}, \qquad [4]$$

must be equal to the total influx of STIL from the cytoplasm

$$k_{10}[PS_c]_{SS} + k_{11}[PS^*{}_c]_{SS} + k_{12}[P^*S_c]_{SS} + k_{14}[P^*S^*{}_c]_{SS} \qquad [5]$$

Since [5] is dependent only the global cytoplasmic concentration, this implies that all compartments have the same flux of STIL degradation and, therefore, PLK4. However, because [4] has two terms, there is a possibility to have an asymmetric state in which not all compartments have identical concentrations if and only if the compartments are locally bistable. Indeed, taking into the consideration [1], at a steady state the concentration of $[P^*S^*{}_i]_{SS} = X_i$ satisfies the following equation:

$$b_1X_i^4 + b_2X_i^3 + b_3X_i^2 + b_4X_i + b_5 = 0, \qquad [6]$$

where all b_i are functions only of the model parameters. Importantly,

$$b_1 < 0 \ and \ \ b_5 > 0$$

regardless of the values of model parameters. This implies that equation [6] can only have either 1 or 3 positive roots. In the first case the model has only one globally stable symmetric state (Figure 5A) and, thus, cannot exhibit symmetry breaking. In the second case, each compartment is bistable as in the model with nonlinear degradation considered in the main text. In this scenario, the system has three symmetric steady states (Figure 5C) of which only the lower symmetric state is accessible from the initial unpopulated state of the centriole as PLK4 and STIL are gradually produced in the cytoplasm.

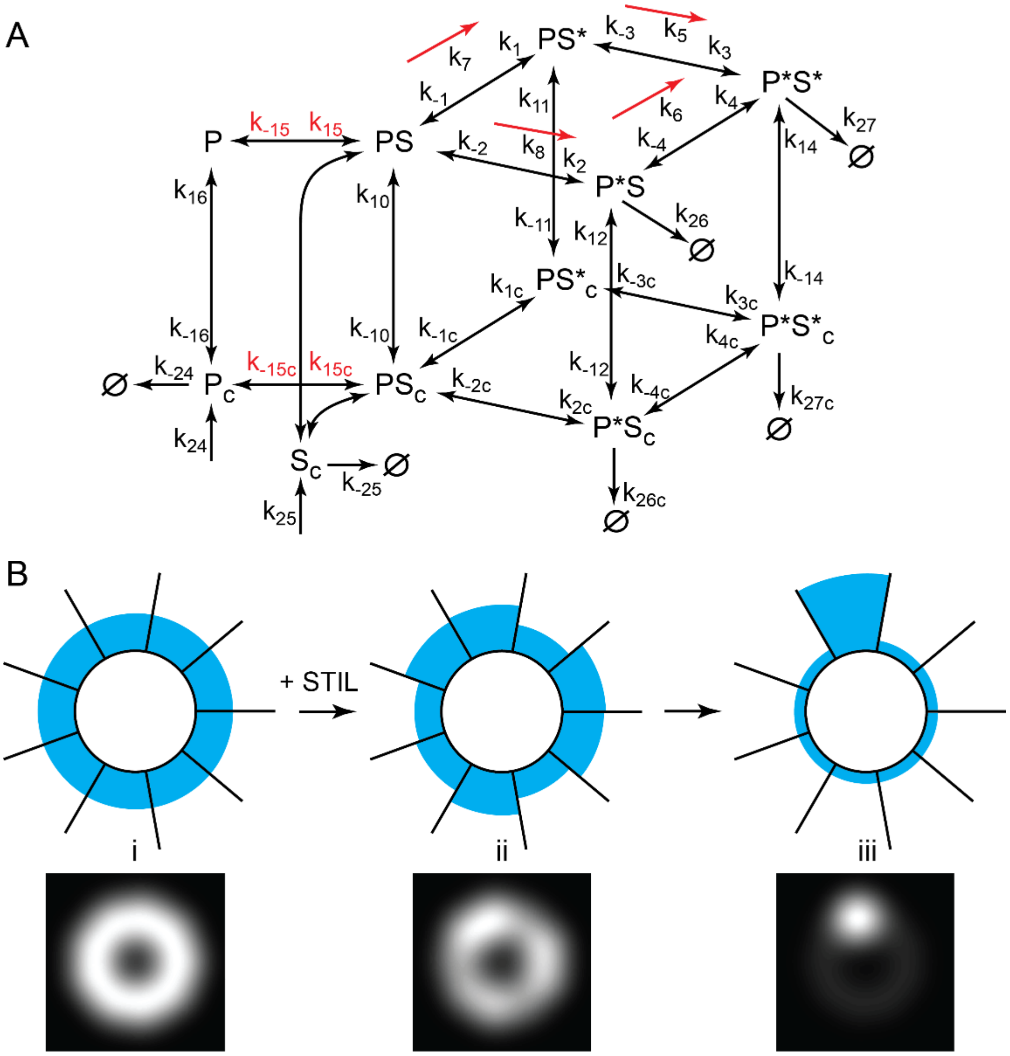

Figure S1, related to Figures 1 and 2. Mathematical formulation of the model. (A) Complete reaction diagram, including the reactions omitted for clarity in Figure 1. Reaction rate constants are shown next to the respective diagram arrows. Association and dissociation rates of complex formation reactions are shown only once (red font). Cross-phosphorylation reactions catalyzed by S*P*, e.g., SP + S*P* → S*P +S*P*, are shown schematically by red arrows. Numeric values of all reaction rates are given in Table S1. All other notations are the same as in Figure 1. (B) Spatial organization of the model. Upper row: model compartments are shown schematically as nine adjacent containers with PLK4 content shown by colored levels (not to scale). Bottom row: respective simulated fluorescence stills. Left to right: i) symmetric “ring” distribution of PLK4 around the centriole prior to the symmetry breaking; ii) just after the onset of symmetry breaking, on average half of compartments attempt to increase their PLK4 content; iii) a characteristic “spot” pattern of the PLK4 distribution upon the resolution of competition between the compartments. In the model, the compartments can exchange their content only via the common cytoplasm. Within the compartments all species are assumed to be spatially uniform (well-mixed).

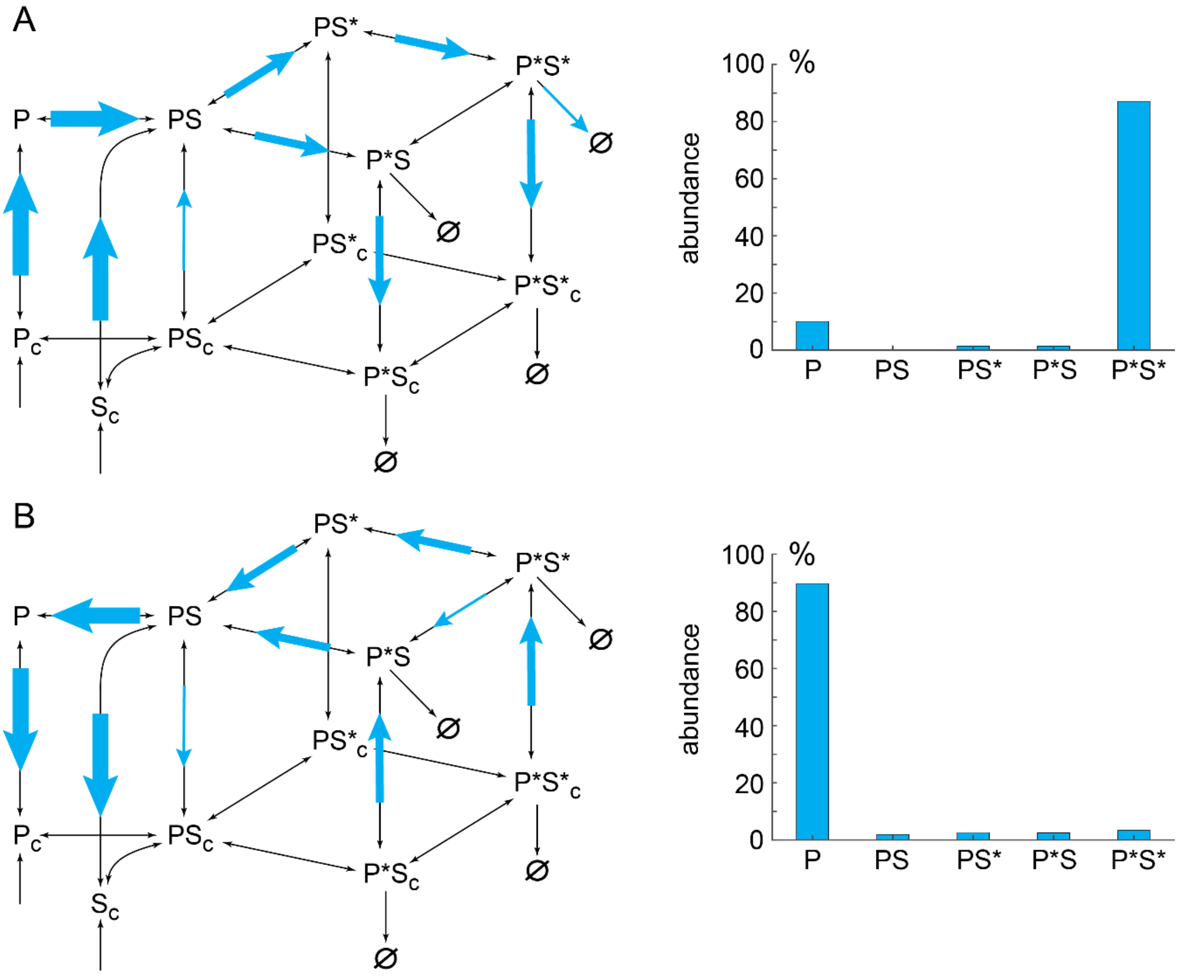

Figure S2, related to Figures 2 and 4. Main reaction fluxes and relative abundances of centriole-bound protein complexes in the winning (A) and losing (B) compartments of the model ~2 hours after the symmetry breaking (5h after the start of simulation). Left column. Relative magnitude of reaction fluxes is schematically shown by the size of colored arrows overlaid over the reaction diagram from Figure 1. The values of fluxes are given in Table S2. Right column. Relative abundances of PLK4 and its complexes with STIL are presented as bar plots.

Supplemental tables

Table S1. Model parameter values.

Rate constant	Value	Reference
$k_1= k_2= k_3= k_4$	1 s^{-1}	this study
$k_{-1}= k_{-2}= k_{-3}= k_{-4}$	1 s^{-1}	this study
$k_5= k_6= k_7= k_8$	10 $(\mu M*s)^{-1}$	this study
$k_{10}= k_{12}=k_{16}$	10 s^{-1}	(Shimanovskaya et al., 2014)
$k_{-10}= k_{-12}= k_{-16}$	0.1 s^{-1}	this study
$k_{11}=k_{14}$	10 s^{-1}	this study
$k_{-11}= k_{-14}$	0.001 s^{-1}	this study
k_{15}	10 $(\mu M*s)^{-1}$	(Arquint et al., 2015)
k_{-15}	1 s^{-1}	this study
$k_{26}=k_{27}$	10^{-4} s^{-1}	(Klebba et al., 2015a; Klebba et al., 2015b)
$k_{1,c}= k_{2,c}= k_{3,c}= k_{4,c}$	1 s^{-1}	this study
$k_{-1,c}= k_{-2,c}= k_{-3,c}= k_{-4,c}$	1 s^{-1}	this study
$k_{15,c}$	10 $(\mu M*s)^{-1}$	this study
$k_{-15,c}$	1 s^{-1}	this study
$k_{24}=k_{25}$	10^{-7} $\mu M/s$	this study
$k_{26,c}=k_{27,c}$	10^{-4} s^{-1}	this study
$k_{-24}=k_{-25}$	10^{-5} s^{-1}	this study
V/V_{cyt}	10^{-4}	this study
a_1	2 * 10^{-5} s^{-1}	this study
a_2	4 * 10^{-5} $(\mu M)^2/s$	this study
a_3	0.1 μM	this study

Table S2. Numerical values of reaction fluxes in the winning and loosing compartments of the model, data for Figure S2. All values are in $10^{-3} \mu M \cdot s^{-1}$.

Reaction flux	Winning compartment	Losing compartment
R16	11.59	-1.302
R15	11.57	-1.328
R1	6.386	-0.575
R2	6.495	-0.9029
R3	6.392	-0.5438
R4	-0.1841	-0.1162
R10	1.353	-0.1875
R11	-0.04353	0.03198
R12	-6.718	0.7893
R14	-5.237	0.6766
R26	0.009834	0.002327
R27	0.5944	0.003047

Supplemental references

Alexiades, V., Amiez, G., and Gremaud, P.-A. (1996). Super-time-stepping acceleration of explicit schemes for parabolic equations. Commun Numer Methods Eng *12*, 31-42.

Arnoldi, W.E. (1951). The principle of minimized iterations in the solution of the matrix eigenvalue problem. Quarterly of applied math *9*, 17-29.

Arquint, C., Gabryjonczyk, A.M., Imseng, S., Bohm, R., Sauer, E., Hiller, S., Nigg, E.A., and Maier, T. (2015). STIL binding to Polo-box 3 of PLK4 regulates centriole duplication. eLife *4*, e07888.

Gillespie, D.T. (2000). The chemical Langevin equation. J Chem Phys *113*, 297-306.

Klebba, J.E., Buster, D.W., McLamarrah, T.A., Rusan, N.M., and Rogers, G.C. (2015a). Autoinhibition and relief mechanism for Polo-like kinase 4. Proceedings of the National Academy of Sciences of the United States of America *112*, E657-666.

Klebba, J.E., Galletta, B.J., Nye, J., Plevock, K.M., Buster, D.W., Hollingsworth, N.A., Slep, K.C., Rusan, N.M., and Rogers, G.C. (2015b). Two Polo-like kinase 4 binding domains in Asterless perform distinct roles in regulating kinase stability. The Journal of cell biology *208*, 401-414.

Lawo, S., Hasegan, M., Gupta, G.D., and Pelletier, L. (2012). Subdiffraction imaging of centrosomes reveals higher-order organizational features of pericentriolar material. Nature cell biology *14*, 1148-1158.

Milo, R., and Phillips, R. (2016). Cell biology by the numbers (New York: Garland Science).

Park, S.Y., Park, J.E., Kim, T.S., Kim, J.H., Kwak, M.J., Ku, B., Tian, L., Murugan, R.N., Ahn, M., Komiya, S.*, et al.* (2014). Molecular basis for unidirectional scaffold switching of human Plk4 in centriole biogenesis. Nature structural & molecular biology *21*, 696-703.

Shimanovskaya, E., Viscardi, V., Lesigang, J., Lettman, M.M., Qiao, R., Svergun, D.I., Round, A., Oegema, K., and Dong, G. (2014). Structure of the C. elegans ZYG-1 cryptic polo box suggests a conserved mechanism for centriolar docking of Plk4 kinases. Structure (London, England : 1993) *22*, 1090-1104.

Sonnen, K.F., Gabryjonczyk, A.M., Anselm, E., Stierhof, Y.D., and Nigg, E.A. (2013). Human Cep192 and Cep152 cooperate in Plk4 recruitment and centriole duplication. Journal of cell science *126*, 3223-3233.

Sonnen, K.F., Schermelleh, L., Leonhardt, H., and Nigg, E.A. (2012). 3D-structured illumination microscopy provides novel insight into architecture of human centrosomes. Biology open *1*, 965-976.
